# Successful treatment of severe interstitial pneumonia by removal of circulating autoantibodies: a case series

**DOI:** 10.1186/s12890-020-01386-2

**Published:** 2021-01-06

**Authors:** Philipp Eller, Holger Flick, Gernot Schilcher, Florentine Moazedi-Fürst, Kathrin Eller, Emina Talakic, Josef Hermann, Yannick Allanore, Horst Olschewski

**Affiliations:** 1 Intensive Care Unit, Department of Internal Medicine, Medical University Graz, Auenbruggerplatz 15, 8036 Graz, Austria; 2 Division of Pulmonology, Department of Internal Medicine, Medical University of Graz, Graz, Austria; 3 Division of Rheumatology, Department of Internal Medicine, Medical University of Graz, Graz, Austria; 4 Division of Nephrology, Department of Internal Medicine, Medical University of Graz, Graz, Austria; 5 Department of Radiology, Medical University of Graz, Graz, Austria; 6 Université Rheumatology A Department, Cochin Hospital, Paris Descartes University, Paris, France

**Keywords:** Interstitial lung disease, Immunosuppression, Vasculitis, Autoimmunity, Radiography

## Abstract

**Background:**

There is only limited clinical data on the benefit of intense immunosuppression in patients with severe interstitial pneumonia associated with autoimmune features or new-onset connective tissue disease.

**Case presentation:**

We here report a series of three consecutive patients suffering from severe interstitial lung disease necessitating endotracheal intubation and mechanical ventilation. The first two patients fulfilled many diagnostic criteria for new-onset antisynthetase syndrome, the third patient for systemic lupus erythematosus. We decided to implement aggressive immunosuppressive strategies in these critically-ill patients including therapeutic plasma exchange, immunoadsorption, cyclophosphamide and rituximab. All three patients improved from respiratory failure, were successfully weaned from the respirator, and eventually dismissed from hospital with ongoing immunosuppressive therapy.

**Conclusion:**

Patients suffering from severe connective tissue disease-associated interstitial lung disease and respiratory failure may benefit from an aggressive immunosuppressive regimen and extracorporeal blood purification with rapid reduction of circulating autoantibodies. The impressive clinical responses in this small case series warrant a controlled clinical trial.

## Background

Currently there are no randomized controlled trials or case–control studies suggesting the efficacy or safety of immunosuppressive agents in patients with interstitial pneumonia with autoimmune features or patients suffering from antisynthetase syndrome [1–7]. We present a series of three consecutive critically-ill patients, who developed severe respiratory failure necessitating endotracheal intubation and mechanical ventilation. All three patients survived their critically illness indicating that our aggressive immunosuppressive strategy aiming at a rapid reduction of circulating autoantibodies might have saved their life.

## Case presentation

### Patient 1

**Fig. 1:**
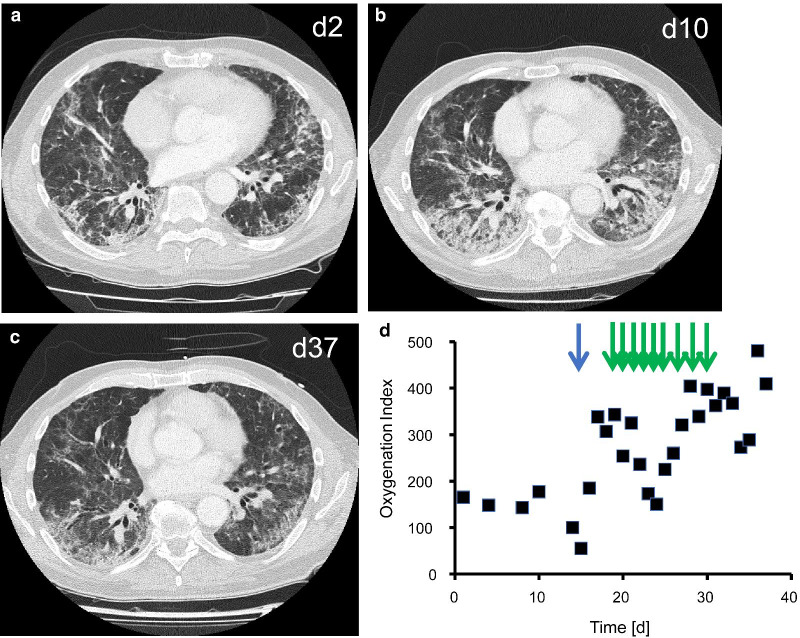
Patient 1. Chest computed tomography scan showing interstitial pneumonia on day 2 (**a**) and day 10 (**b**) with mainly axial distribution, subpleural consolidations and no honeycombing. Improvement in the chest computed tomography scan on day 37 (**c**). There was a close temporal association of the initiation of therapeutic plasma exchange on day 14 (blue arrow), and the nine immunoadsorptions (green arrows from day 19 until day 30) with recovery of oxygenation index (closed squares) (**d**)

This 60-year-old male had progressive dyspnea for 4 weeks, general weakness, and involuntary loss of ten kg body weight over the last months. His past medical history was largely unremarkable. He had received caspofungin (70 mg o.d.) medication over the last 3 weeks without any clinical improvement. This medication was initiated because of a positive PCR result for *Pneumocystis jirovecii* in the bronchial lavage fluid, which was obtained during an outpatient bronchoscopy performed 26 days ahead of the admission to hospital. In parallel, the serum levels of beta-d-glucan had been elevated to 219 pg/ml (normal range < 80 pg/ml). He had a full time job as electrician and had worked with mineral wool for the last six months. In the initial clinical examination, there were rales on both lungs and elevated temperatures up to 37.4 °C, but neither myopathy nor other signs of connective tissue disease. Chest computed tomography scan on day 2 (Fig. 1a) showed bilateral interstitial lung disease with mainly axial distribution, subpleural consolidations and no honeycombing, as they typically occur in connective tissue disease-associated interstitial lung disease [8]. The laboratory analysis revealed elevated PL-12 antibody titers of 110 U/mL and anti-Ro52 titers of 26 U/mL (normal for both < 10) suggestive of antisynthetase syndrome, but no elevations of creatine kinase and no mechanics hands. The patient received prednisolone (1 mg/kg body weight o.d.). Unfortunately, dyspnea, and hypoxia continued to worsen and infiltrates in chest computed tomography scan on day 10 showed severe deterioration (Fig. 1b). The patient developed progressive respiratory failure necessitating non-invasive mechanical ventilation and finally endotracheal intubation. The control PCR from the bronchial lavage fluid performed immediately after the endotracheal intubation was negative for *Pneumocystis jirovecii,* and beta-D-glucan serum levels were in the normal range, thus essentially excluding an ongoing infection with *Pneumocystis jirovecii.* Due to the rapid clinical deterioration, we decided to implement an aggressive immunosuppressive therapy including removal of circulating autoantibodies by means of a series of plasma exchange treatments followed by a long-term immunosuppression with cyclosporin A. We calculated the plasma volume from height, weight and hematocrit, and used fresh frozen plasma as replacement fluid. As the patient developed a transfusion-related acute lung injury after the second plasma exchange therapy, we switched from plasma exchange treatment to daily immunoadsorption and aimed in parallel for cyclosporin A blood levels of 100–200 ng/mL. We used the peptide GAM as sorbent to specifically remove immunoglobulins G [9]. Under this immunosuppressive regime with a series of nine immunoadsorptions and concomitant T-cell inhibition with cyclosporine A, the patient gradually improved, was extubated after six days, and discharged from the intensive care unit with clear regression of interstitial pneumonia in the computed tomography scan on day 37 (Fig. 1c). There was a close temporal association of initiation of immunosuppression with recovery of oxygenation index (Fig. 1d). We regularly screened the patient for viral superinfections. On the day of admission to the ICU and during the previous weeks, there was no sign for a reactivation of cytomegalic virus infection neither in blood nor in bronchial lavage fluid. However, we found slightly elevated titers for cytomegalic virus in blood (460 copies/ml) on day 22, when the patient was successfully weaned from the respirator. Six days later and after the initiation of sulfametrol/trimethoprim and ganciclovir therapy (5 mg/kg body weight o.d.), cytomegalic virus was no longer detectable. The patient was dismissed from hospital after 56 days with long-term oxygen therapy of 2L/min for at least 16 h/day. The follow-up controls after 6, and 12 months showed a stable disease with low anti-PL12 antibody titers (34–45 U/mL) under ongoing immunosuppressive therapy with prednisolone and cyclosporine A. Prednisolone and long-term oxygen therapy were both terminated after 12 months.

### Patient 2

**Fig. 2:**
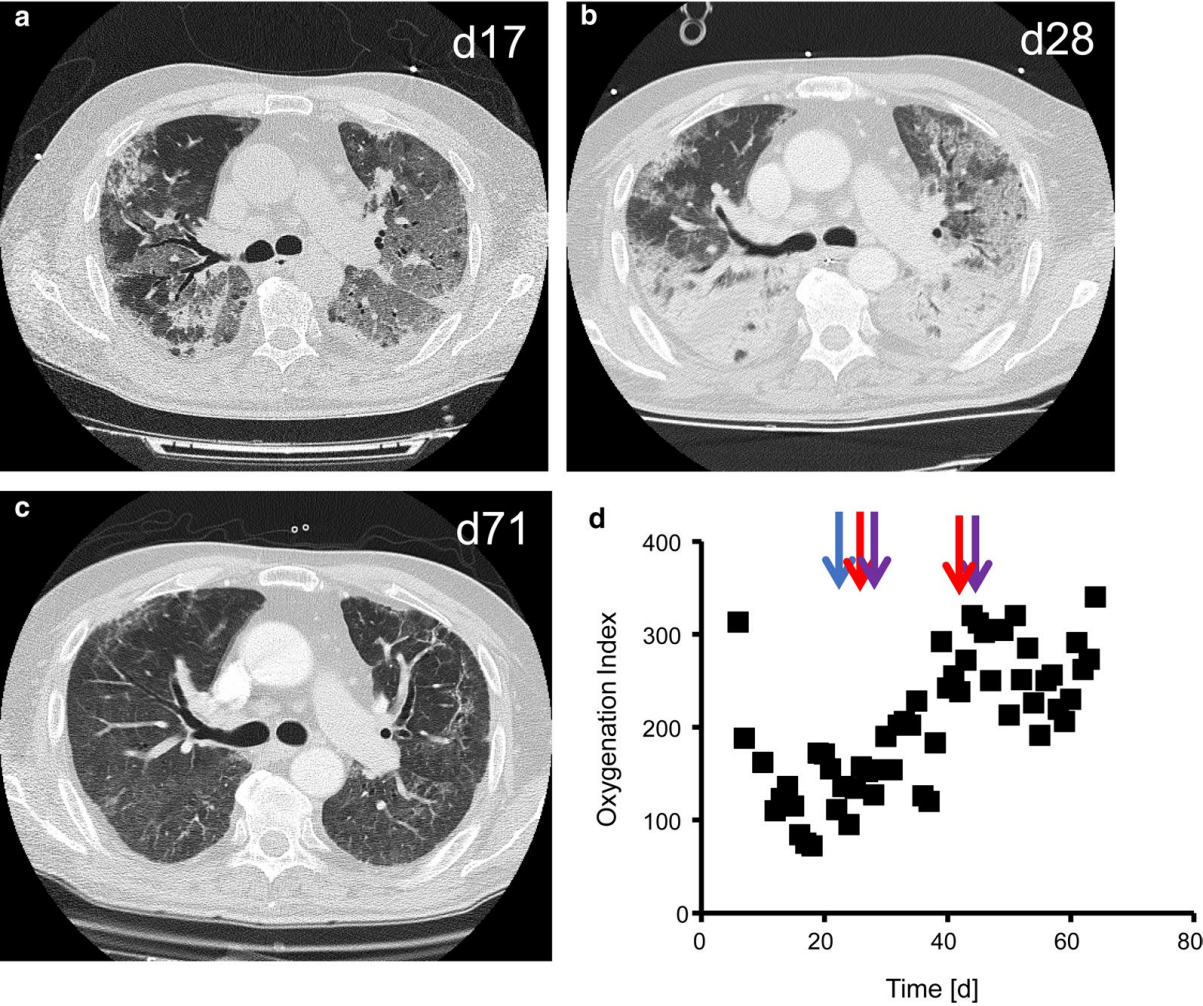
Patient 2. Chest computed tomography showing progressive interstitial pneumonia on day 17 (**a**) and day 28 (**b**) with dense and partly consolidated infiltrates, fine reticular pattern and no honeycombing. Dramatic improvement in the chest computed tomography scan on day 71 (**c**). There was a close temporal association of the initiation of therapeutic plasma exchange on day 24 (blue arrow), and the infusion of cyclophosphamide (red arrow) and rituximab (violet arrow) on day 28 and 42 with recovery of oxygenation index (closed squares) (**d**)

This 59-year-old male patient suffered from progressive dyspnea, cough without expectorations, and symmetric proximal muscular weakness for 2 weeks. His medical history was unremarkable. He had a full time job as metalworker and took no premedication. A few days before admission, he experienced pain in the small joints of hands and feet. The initial clinical examination revealed rales on both lungs and fever up to 38.3 °C. The wrists and finger joints were not swollen. Chest x-ray showed bilateral infiltrates, and laboratory analysis revealed elevated creatinine kinase blood levels of 4822 U/L (normal < 170 U/L), and positive anti-Jo-1 antibody titers of 80 U/mL and anti-Ro52 titers of 104 U/mL (normal for both < 10 U/mL) suggestive of antisynthetase syndrome. The patient received prednisolone therapy (1 mg/kg body weight o.d.), low-dose cyclosporine A (50 mg t.d.), and intravenous immunoglobulins (0.4 g/kg body weight o.d.). Both dyspnea and radiological features rapidly worsened under this treatment as shown by the dramatic deterioration in the chest computed tomography scan on day 17 (Fig. 2a) which revealed dense and partly consolidated interstitial pneumonia with mainly subpleural consolidations, fine reticular pattern and no honeycombing. After 18 days in hospital, the patient was transferred to the intensive care unit where immediate intubation and mechanical ventilation was necessary. As we did not find any clue for opportunistic infections in the extensive microbiological workup of blood and bronchial lavage samples and the chest computed tomography scan on day 28 showed extensive progression of disease (Fig. 2b), we decided to implement removal of circulating autoantibodies and an aggressive immunosuppressive therapy including a series of five plasma exchange treatments followed by rituximab (1000 mg) and cyclophosphamide (10 mg/kg body weight) infusions on day 28. We terminated the initial low-dose cyclosporine A treatment. As the patient improved functionally and morphologically in the computed tomography scan of the lungs, we repeated the infusion with rituximab and cyclophosphamide after 2 weeks. The patient was extubated on day 48. The chest computed tomography scan on day 71 showed complete resolution of the interstitial pneumonia (Fig. 2c). There was a close temporal association of the initiation of immunosuppressive treatment and functional recovery of oxygenation index (Fig. 2d). The patient was discharged from hospital on day 78 with long-term oxygen therapy of 2L/min > 16 h/day. The follow-up controls after two months showed a stable disease without any need of long-term oxygen therapy, low anti-Jo-1 antibody titers (25 U/mL) and low anti-Ro52 antibody titers (26 U/mL) under ongoing immunosuppressive therapy with rituximab and prednisolone.

### Patient 3

**Fig. 3:**
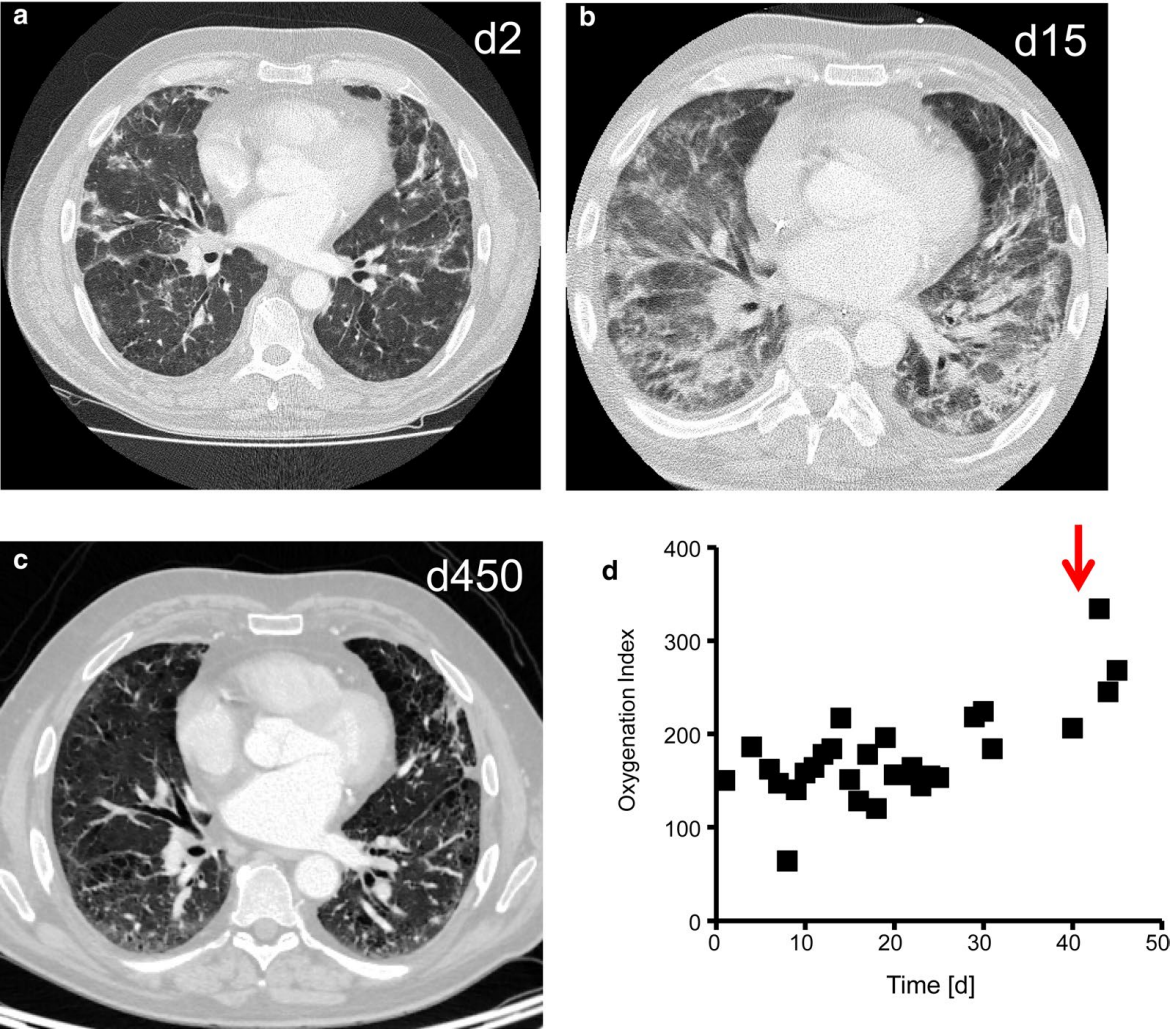
Patient 3. Chest computed tomography showing interstitial pneumonia on day 2 (**a**), day 15 (**b**). Immunosuppression with cyclophosphamide (red arrow) was initiated on day 43, causing impressive clinical improvement. A control chest computed tomography was performed after 15 months (**c**). There was a close temporal association of the initiation of cyclophosphamide (red arrow) on day 43 with recovery of oxygenation index (**d**)

This 60-year-old male had suffered from progressive dyspnea over the last two years. He had retired from work as a pavior at the age of 59 years. In the initial clinical examination, there were rales over the lungs, and indolent erythematous plaques in the face, that had exacerbated after exposure to sunlight. Chest computed tomography on day 2 showed lung emphysema with mainly subpleural interstitial consolidations (Fig. 3a). Laboratory analysis revealed positive fine-speckled anti-nuclear antibodies with a titer of 1:1280 and anti-Ro60 antibodies of 17 U/mL (normal < 10 U/mL). Despite non-invasive ventilation and escalation of the prednisolone therapy to 1 mg/kg body weight o.d., the patient rapidly deteriorated and was intubated on day 8 due to hypercapnic respiratory failure. We tried to wean the patient from the respirator after seven days, but had to re-intubate him on day 18 due to a hypercapnic weaning failure (Fig. 3b). The patient was finally extubated on day 24. As the patient did not substantially improve under prednisolone treatment (1 mg/kg body weight o.d.), we escalated immunosuppressive therapy by giving an infusion of cyclophosphamide on day 43 (500 mg every 4 weeks). The patient slowly improved under this immunosuppressive treatment regime (Fig. 3c). After 55 days, he was dismissed from hospital with long-term oxygen therapy of 2L/min > 16 h/day. The follow-up controls showed reduced requirement of long-term oxygen therapy, low anti-nuclear antibody titers (1:160), and no interstitial pneumonia in the control chest tomography scan after 15 months under ongoing immunosuppressive therapy with mycophenolate mofetil (1000 mg t.d.) and prednisolone (Fig. 3d).

## Discussion and conclusions

We report of three consecutive patients suffering from connective tissue disease-associated interstitial lung disease and severe respiratory failure in a desperate situation who obviously benefited from aggressive immunosuppressive regimens. The clinical responses were evident in sequential computed tomography scans, in the functional recovery as given by blood gas analyses, and by the physical status of the patients. All of them recovered to a status similar to the time before exacerbation of their disease. Interestingly, disease activity was closely correlated with autoantibody titers in all patients. In the cases with features of antisynthetase syndrome, we aimed to remove autoantibodies from the blood as quickly as possible using plasmapheresis and/or immunoadsorption.

Of course, this small dataset does not allow making therapy recommendations. However, the clinical responses were so impressive that we decided to report it to the community. Our previous success rate in similar patients where we did not apply such aggressive therapy had been quite poor. Therefore, this series of consecutive successful cases adds further evidence to similar previous reports and suggests that in special clinical constellations only removal of circulating autoantibodies may provide a realistic chance for success [10–12].

Treatments such as plasma exchange, immunoadsorption, and cyclophosphamide with rituximab or cyclosporine A could be considered in similar cases of severe progressive interstitial lung diseases with no response to corticosteroid treatment, particularly in patients with antisynthetase syndrome. Our case series also shows that discrimination between interstitial pneumonia with autoimmune features and interstitial lung disease due to new-onset connective tissue disease may be difficult. However, such discrimination seems to have limited therapeutic implications in critically-ill patients.

Interestingly all three consecutive cases were males around 60 years of age who had been very active in their job as craftsmen and in two cases had not had any signs and symptoms of disease until few weeks before decompensation. Maybe this constellation is associated with a higher success rate for aggressive therapy than other clinical constellations. On the other hand, two of our patients had elevated titers of anti-Ro-52 antibodies, which constitutes a potential risk marker for worse pulmonary outcomes in interstitial lung disease [13, 14].

Our experience warrants a controlled clinical trial testing the safety and efficacy of removal of circulating autoantibodies in patients suffering from connective tissue disease-associated interstitial lung disease with severe respiratory failure.

## Data Availability

The datasets used and/or analysed during the current study are available from the corresponding author on reasonable request.
